# *Callosobruchus maculatus* males and females respond differently to grandparental effects

**DOI:** 10.1371/journal.pone.0295937

**Published:** 2023-12-22

**Authors:** Azam Amiri, Ali R. Bandani

**Affiliations:** 1 College of Geography and Environmental Planning. University of Sistan and Baluchestan, Zahedan, Iran; 2 Department of Plant Protection, College of Agriculture and Natural Resources, University of Tehran, Karaj, Iran; University of Vienna, AUSTRIA

## Abstract

In this study, we used the cowpea weevil *Callosobruchus maculatus* (Coleoptera: Chrysomelidae) and two essential oils (mint and rosemary) to investigate the effect of the parents (F0) exposure to a sublethal dose of essential oil on grand offspring (F2) encountering the same essential oil. Then we evaluated biological parameters, including immature development time, sex ratio, adult emergence, egg number, egg hatch, longevity, and mating behaviors in three generations (F0, F1, and F2). Results showed when F0 experienced essential oil in the embryonic stage, parental and grandparental effects were more severe than adulthood experiences. Also, grandparental effects increased or decreased reactions of F2 generation when faced with a similar essential oil, depending on grand offspring sex. For example, when grandparents experienced rosemary essential oil in the embryonic stage, they produced more tolerant female grand offspring with a better ability to cope with the same essential oil (increased adult longevity and egg number). However, male grandoffspring were more sensitive (had a higher mortality percentage and less copulation success). Grandparental effects of exposure to mint essential oil diminished female grand offspring longevity and improved male copulation behavior parameters such as increased copulation duration and decreased rejection by females. In all, grandparental effects were different in male and female grand offspring based on the essential oil type experienced by F0.

## Introduction

Transgenerational effects are nongenetic environmental impacts transmitted across generations, causing phenotypic variation [1, 2]. Transgenerational effects send information from ancestors (senders) to descendants (receivers) through various mechanisms, including epigenetic, morphological, physiological, neurological, and/or behavioral alterations [3–5]. They can last many generations, but the most well-studied are the parental effects that mothers (maternal) and fathers (paternal) pass on to their progeny [6–8]. Parental effects, or nongenetic information transmission through generations, can considerably affect offspring physiology, morphology, behavior, and life-history features [9–11].

Offspring carry this information from their parents, modulate/update it through their experience, and pass it on to their offspring [12]. While parental effects have been extensively studied, we have limited knowledge of how parental or grandparental effects interact across generations in males and females [4, 13, 14].

Transgenerational effects are thought to be evolutionarily beneficial for offspring [4, 13]. Transgenerational effects may be adaptive in both matching and non-matching ancestor and offspring environments. For example, in *Amblyseius swirskii* mites, grandparental and parental feeding experiences mix with early-life learning to generate adaptive foraging phenotypes [12]. We have shown in our previous study that the interval between adult cowpea weevil stress exposures and following mating influences the quality of parental and grandparental effects [15].

Also, sex-selective transgenerational plasticity passed down through specific lineages or to exclusively male or female progeny may be advantageous if it permits previous generations to fine-tune future generations’ phenotypes in response to sex-specific life-history tactics. However, the effects of the sex of offspring or grand offspring on parental effects are underdocumented [16].

Another essential and underdocumented feature that may impact parental or grandparental effects is the life stage of senders (parents or grandparents) when experiencing a particular event or environment. Early life may be particularly vulnerable to the transmission of environmental effects between generations [17]. Early life experiences have substantial and long-lasting consequences for adult phenotypes, especially behaviors [18–20]. Conditions experienced during early-life development may also affect future generations [21]. Transgenerational effects were demonstrated to improve offspring performance in both stressful and non-stressful situations [22].

There is evidence that organisms that are exposed to stressful situations, whether abiotic or biotic, can prime gene expression in their offspring to help them cope with stress better [23, 24]. Sublethal pesticide exposure can cause stress and contribute to more significant phenotypic variation [25]. Insecticides and other xenobiotics can modify the status of DNA methylation in arthropods, and these epigenetic modifications can last for several generations [5, 26].

Essential oils represent an example of the xenobiotics mentioned above. Essential oils are concentrated hydrophobic liquids that contain volatile compounds from some plants. They quickly evaporate at room temperature. Plant essential oils and active ingredients have the potential to show remarkable bioactivity against a variety of insects, including the cowpea weevil *Callosobruchus maculatus*, which has repellent, contact, fumigant toxicity, ovicidal activity, oxidative stress, or susceptibility-enhancing properties [27–33].

Essential oil’s chemical composition is linked to its toxicity to insects. The bioactivity of an essential oil in a pest insect is related to not only the major constituent(s) but also synergy among constituents [34]. Plant essential oils are composed of different constituents that result in various responses. Menthol and menthone are two dominant constituents of mint essential oil (together, 65–85% by weight). Major constituents of rosemary essential oil are 1,8-cineole (52%), α-pinene (10%), camphor (9%), and β-pinene (8%) [34].

Different studies [35–38] have reported that mint and rosemary essential oils are toxic to *C*. *maculatus* and thus considered environmental stressors. For example, the insecticidal activity of rosemary essential oil and intergenerational resistance transmission have been reported in *C*. *maculatus* [38]. Also, the insecticidal effects of various *Mentha* species essential oils were studied against *C*. *maculatus* and different pests [39].

*C*. *maculatus* (2.0–3.5 mm long) is a significant pest of cowpeas and other legumes distributed throughout the tropics and sub-tropics. Adults are facultative aphagous short-lived insects (around one to two weeks) that do not need to feed or drink [31, 40]. After mating, female *C*. *maculatus* deposits eggs on the legume seed surface. The larvae enter the seed after hatching and feed exclusively within a single grain, so the larval stage and pupation take place within the grain, whereas the adult leaves the seed following emergence. If they come across a female, newly-emerged adult males normally copulate right away [31, 40, 41].

In this study, we aim to investigate: (i) the effect of the parent generation (F0) exposure to essential oil on their grand offspring (F2 generation) reaction -more susceptible or more resistant- when experiencing the same essential oil; (ii) to understand if grand offspring sex makes any differences in the grandparental effects they received; (ⅲ) to document which stage/s of grandparents (embryo or adult) can better transfer grandparental effects to grand offspring. Thus, we used *C*. *maculatus* and two plant-essential oils (mint and rosemary) to test these hypotheses. Therefore, to achieve our aim, we used two stages of the insects, including embryo (egg) and adult, in the test experiments.

## Methods

### Plant materials and essential oil extraction

We collected leaves of *Mentha spicata* (mint) and *Rosmarinus officinalis* (rosemary) from Zahedan, Sistan and Baluchestan province, Iran (Latitude: 29.4519, Longitude: 60.8842, Elevation above sea level: 1352 m). The leaves were air-dried at room temperature for one week and then hydrodistilled to extract the essential oils.

After grinding dried leaves (300 g), essential oils were extracted by hydrodistillation in a Clevenger apparatus for 3 hours. Finally, water was eliminated by anhydrous sodium sulfate, and the extracted oil was stored in a dark box in a refrigerator at 4°C.

### Insect colony

The *C*. *maculatus* population used for this study was derived from the Plant Protection Department of the University of Shiraz, Fars province, Iran (Latitude: 29.5926, Longitude: 52.5836, and Elevation above sea level: 1585 m).

The strain of *C*. *maculatus* was reared on black-eyed beans (*Vigna unguiculata*) for five generations before beginning the experiments under laboratory conditions at 30±1°C, 50±5% RH, and 16L: 8D photoperiod.

**Fig 1:**
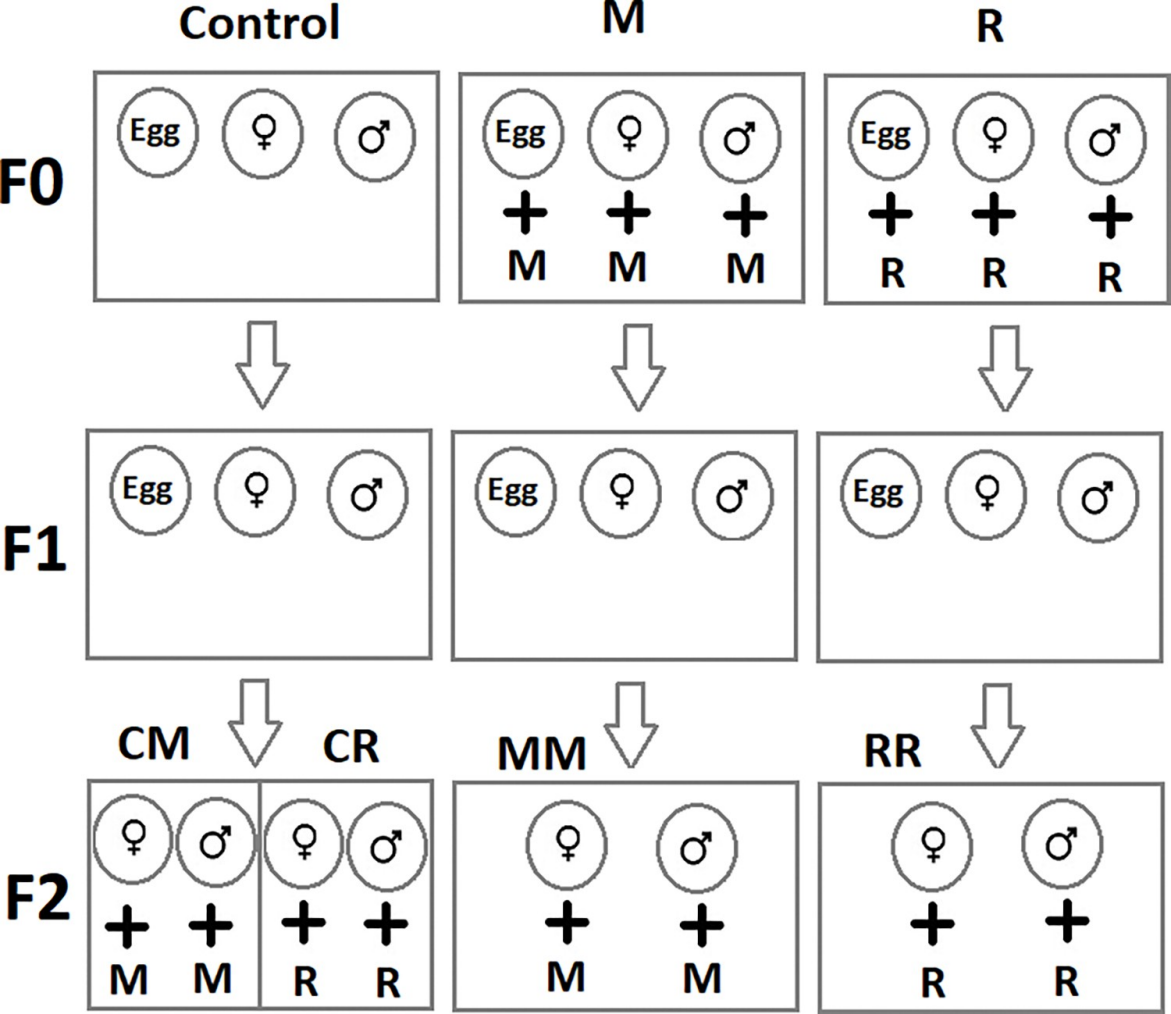
Schematic figure of the experimental setup. Control: No essential oil exposed insects; R: Rosemary essential oil exposure; M: Mint essential oil exposure; CR: F2 generation of control that treated with rosemary essential oil; RR: F2 generation of R treatment that treated with rosemary essential oil; CM: F2 generation of control that treated with mint essential oil; MM: F2 generation of M treatment that treated with mint essential oil.

We used eggs (<24 h-old) and the newly emerged (<24 h-old) virgin adult males and females of *C*. *maculatus* to set up the experiments. Virgin adults were collected by placing each bean with egg on the surface in a separate Petri dish (60 mm diameter) and eliminating adults as soon as they emerged. In this study, we used beans with only one egg on them. A schematic diagram of the experimental setup is presented in Fig 1.

### F0 essential oil exposure

We used 380 eggs (<24 h-old; 80 for control, 150 for mint, and 150 for rosemary essential oils) and 600 newly emerged (<24 h-old; 180 for control, 210 for mint, and 210 for rosemary essential oils) virgin adults of *C*. *maculatus* to set up the F0 stress exposure test. Each Petri dish (diameter 60 mm) separately contained either ten beans with one egg on the surface of each bean, ten freshly emerged virgin males, or ten freshly emerged virgin females. The Petri dishes’ lids were covered with filter paper from the inside. Based on an initial dose-setting experiment [31], we chose a sublethal dose of essential oils after 24 hours of fumigant toxicity to employ essential oils as environmental stressors. Then, we applied 0.7 and 1 μl of *M*. *spicata* and *R*. *officinalis* leaf essential oils (corresponding to essential oil concentrations of 26.5 and 37.8 μl/l_air_) to the filter paper pieces. Then, the Petri dishes’ edges were sealed with parafilm. After 24 hours of incubation, the insects and eggs were transferred to other Petri dishes with a net lid. The control group was not exposed to any essential oil.

### Monitoring and record-keeping

For the embryo essential oil exposure (EEO), each treated bean with a single egg on it was individually placed in a Petri dish. We monitored the beetle’s hatching percentage after the essential oil exposure. Successful hatching was confirmed based on the subsequent production of color. Hatched eggs turned whitish, while unhatched eggs remained translucent and glossy [42]. We recorded F0 immature development time (egg to adult emergence), adult emergence, and longevity every day. The monitoring continued until the death of the last individual.

After the adults’ emergence, we did 42 copulation tests using 42 virgin males and 42 virgin females (two by two in a separate Petri dish and only once for each couple). After mating, 12 insects were transferred to a Petri dish (six males and six females) and checked daily to record their survival.

We transferred all eggs from the first-day post-copulation to other Petri dishes and used them as F1. In other words, we used 2867 eggs for the EEO test (773, 1046, and 1048 eggs for C, R, and M, respectively. C: Control; R: Rosemary essential oil treatment; M: Mint essential oil treatment) for F1 formation. We put each bean with the eggs on the surface separately in Petri dishes and recorded F1 immature developmental time, adult emergence, sex ratio, and adult longevity egg number and hatch percentage. When the larvae grew and became imagoes, we randomly used 84 newly emerged (<24 h-old) virgin males and females from each treatment for the F1 copulation test and used them as the parents to produce the next generation (F2). In sum, we used 252 insects as the parents of EEO, and all the eggs of the first day of these parents were used to form the F2 generation.

For the adult essential oil exposure treatment (AEO), we let treated insects mate (two by two) an hour after essential oil exposure termination. Eighty-four insects for each treatment of the copulation test (C, R, and M) were used (in sum, 252 for all treatments of the AEO test). Then we placed six males and six females in a Petri dish for six replications. All eggs of the first day post-copulation (in total, 2924 eggs for the AEO test, including 1233, 793, and 898 eggs for C, R, and M, respectively) were collected for the F1 formation.

F0 and F1 biological parameters were documented, like the EEO test. F2 was set up similarly to the F1 establishment.

### F2 essential oil exposure

We utilized F2 newly emerged (<24 h-old) virgin adults of control, R, and M treatments of EEO, and AEO tests to study the effect of F0 essential oil exposure on F2 performance where adults were challenged with the same essential oil as what F0 experienced. We used rosemary and mint essential oils for the F2 of the control grandparents (CR and CM, respectively) and the F2 of essential oil-exposed grandparents (RR and MM, respectively). Essential oil volumes were the same as in the F0 treatment. After 24 hours of incubation, we did a copulation test an hour after essential oil exposure. We then recorded biological parameters the same as the F0 tests for the F2 generation.

### Copulation test

We used virgin adults (<24 h-old) from all treatments (EEO: C, M, R, CM, MM, CR, and RR; and AEO: C, M, R, CM, MM, CR, and RR) to conduct the mating test for all the experiments. Essential oils-treated males mated with treated females and control males mated with control females. We put a male in a Petri dish, immediately transferred a female, and gave them ten minutes to begin copulation. Then, we recorded mating traits (mating latency, the beginning of kicking, kicking duration, and termination of copulation) using a digital chronometer [31].

Mating latency shows the time interval between the addition of a female insect to a male Petri dish and the commencement of copulation by the male insect. The kicking phase start represents the time interval between the copulation beginning and the female kicking struggle to terminate copulation. Kicking duration shows the time when a female keeps kicking a male, and finally, copulation duration is the interval between the commencement of mating and its termination.

When a male did nothing up to ten minutes from the start of the copulation test, we considered it a male with no tendency. We named it a rejected male when females actively rejected the male copulation effort. We performed all mating tests during the light phase from 13:00 to 15:00. We repeated the experiment 42 times for each treatment (with a new male and female in each mating encounter).

### Statistical analysis

We performed Linear Mixed Model analyses [43] using SPSS version 27.0 to deal with non-independencies in our data, including a mixture of fixed and random effects as predictor variables. We estimated the relationship between treatments and different biological parameters of F0, F1, and F2 (immature development time, adult longevity, mating latency, kicking phase start, kicking duration, copulation duration, egg number, and hatching percentage). We modeled Petri dishes as random effects to account for the non-independence of data points and reduce the probability of false positives and negatives. We also considered the treatment response as a dependent variable. The Chi-square goodness-of-fit tests were done for comparison of percentages of successful mating, rejected males, and males with no tendency. Statistical significance was established at P<0.05. The values are means±SE. We also fitted multivariate models using the multivariate General Linear Model with Tukey’s post hoc multiple comparisons, Bonferroni confidence interval adjustment, Wilks’ Lambda multivariate test, and Levene test for equality of variances (S1 Table in S1 File).

## Results

### Biological parameters of F0 generation in embryonic exposure

A schematic diagram of the experimental setup is presented in Fig 1. Also, an overview of factors that influence the *C*. *maculatus* phenotype is summarized in Table 1.

**Table 1:** Overview of factors that influence the *C*. *maculatus* phenotype.

Factor	Generation	Sex	Stressors	Life stage
Phenotype				
Adult longevity	F0	-	*	*
	F1	-	-	-
	F2	*	*	*
Development time	F0	-	-	-
	F1	-	-	-
	F2	-	-	-
Egg number	F0	*	*	*
	F1	*	-	-
	F2	*	*	*
Egg hatch	F0	-	-	-
	F1	-	-	-
	F2	-	-	-
Male Success	F0	*	-	-
	F1	*	-	-
	F2	*	-	-
Rejected male	F0	*	*	*
	F1	*	-	-
	F2	*	*	*
Male with no tendency	F0	*	*	-
	F1	*	-	-
	F2	*	*	*
Mating latency	F0	*	-	-
	F1	*	-	-
	F2	*	*	*
Kicking phase start	F0	*	*	*
	F1	*	-	-
	F2	*	*	*
Kicking duration	F0	*	*	*
	F1	*	-	-
	F2	*	*	*
Copulation Duration	F0	-	*	*
	F1	-	-	-
	F2	-	*	*

Asterisks represent statistical significance (P<0.05).

**Fig 2:**
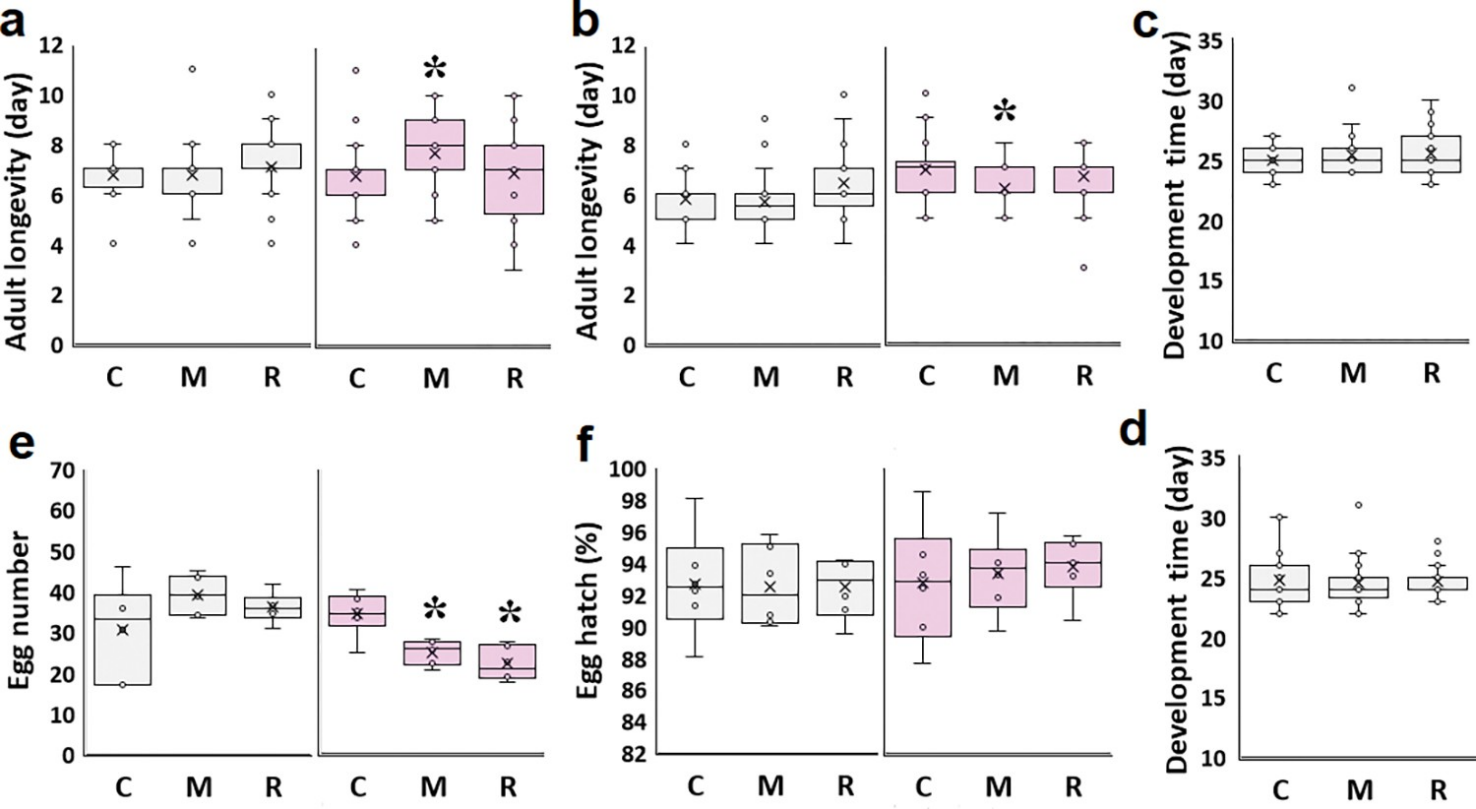
Effect of mint and rosemary essential oil exposure on different biological parameters of *C*. *maculatus* F0 generation. a: Female adult longevity (day); b: Male adult longevity (day); c: Female immature development time (day); d: Male immature development time (day); e: Egg number; f: Egg hatch (%); C: No essential oil exposure; M: Mint essential oil exposure; R: Rosemary essential oil exposure. Grey and pink colors presented EEO and AEO conditions. Asterisks represent statistical significance (P<0.05). The mean is indicated by a cross and dots (outliers) represent data more or less than normal.

Embryo essential oil exposure (EEO) did not alter insect biological parameters, including immature development time, male and female adult longevity, egg number, and hatch percentage (Fig 2A–2F).

### Mating behaviors of the F0 generation in embryonic exposure

**Fig 3:**
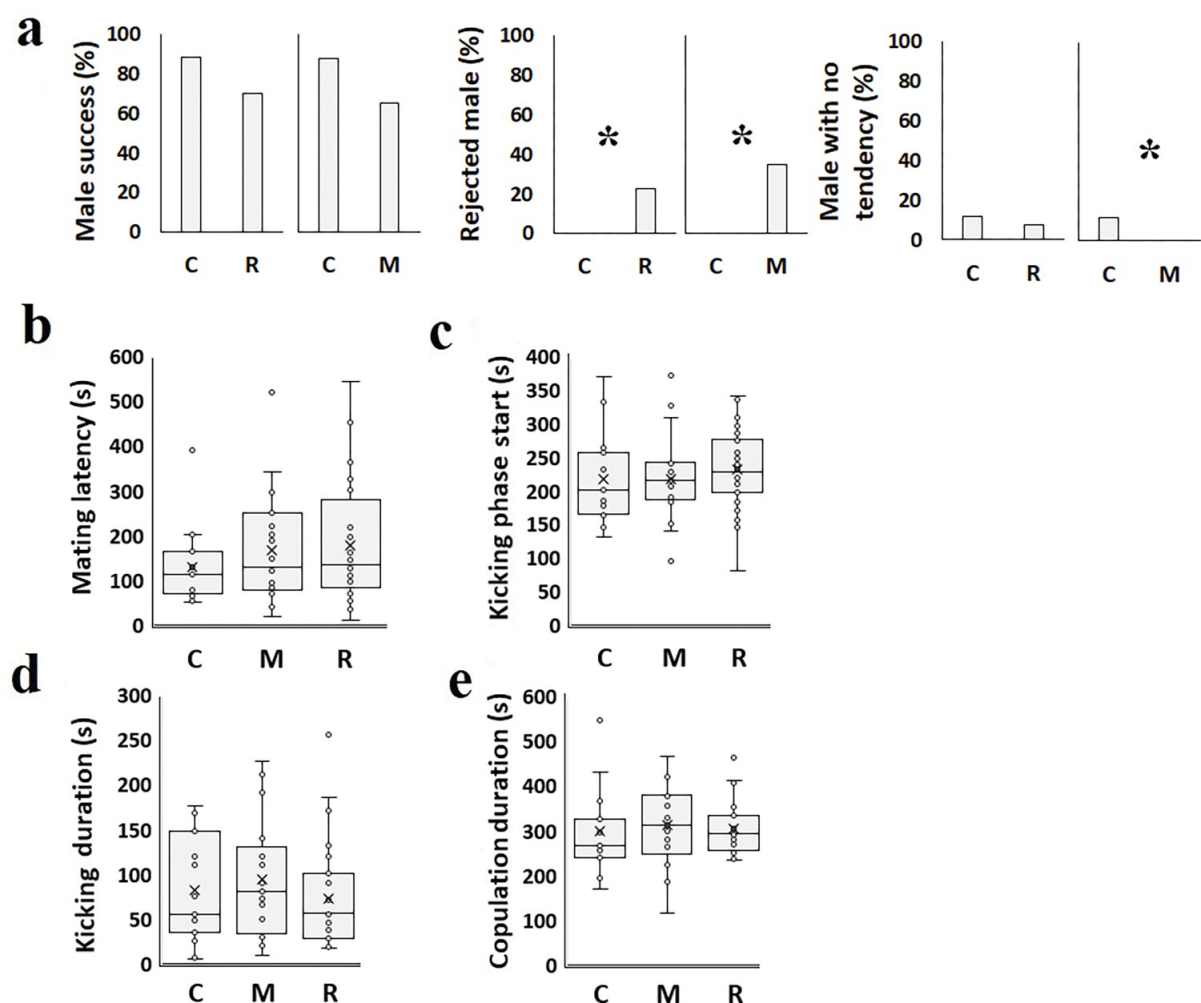
Effect of *C*. *maculatus* exposure to rosemary and mint essential oil in the embryonic stage on copulatory traits of F0 generation. a: Successful mating (%); b: Mating latency (s); c: Kicking phase start (s); d: Kicking duration (s); e: Copulation duration (s). C: No essential oil exposure; M: Mint essential oil exposure; R: Rosemary essential oil exposure. Asterisks represent statistical significance (P<0.05).

EEO affected some copulatory behaviors in adulthood. For example, the females-rejected-males’ percentage declined following mint oil exposure (M) (34.4%, χ^2^ = 31.114; df = 1; P< 0.001) and rosemary essential oil exposure (R) (22.5%, χ^2^ = 20.167; df = 1; P< 0.001) compared to control (0%; Fig 3A). However, some mating behaviors were not influenced, including mating latency, kicking phase start, kicking duration, and copulation duration (Fig 3B–3E).

### Biological parameters of the F0 generation in adult exposure

In the AEO experiment, where insects were exposed to the essential oil in adulthood, mint essential oil (M) increased female adult longevity compared to control females (7.6±0.3 days, df = 103, t = 2.5, P = 0.013, and 6.7±0.2 days, t = 25.5, respectively). However, male adult longevity decreased (6.2±0.3 days, df = 103, t = -2.3, P = 0.033, and 6.9±0.2 days, t = 32.2, for M and C, respectively).

Also, in AEO, both mint and rosemary essential oil-treated females deposited fewer eggs than control (24.9±2.4 eggs, df = 15, t = -3.8, P = 0.002, 22.2±2.4 eggs, t = -5.0, P<0.001, and 34.2±1.7 eggs, t = 19.8, respectively, Fig 2E). The values are means±SE. The hatching percentage of the eggs was not altered by essential oil exposure (Fig 2F).

### Mating behaviors of the F0 generation in adult exposure

**Fig 4:**
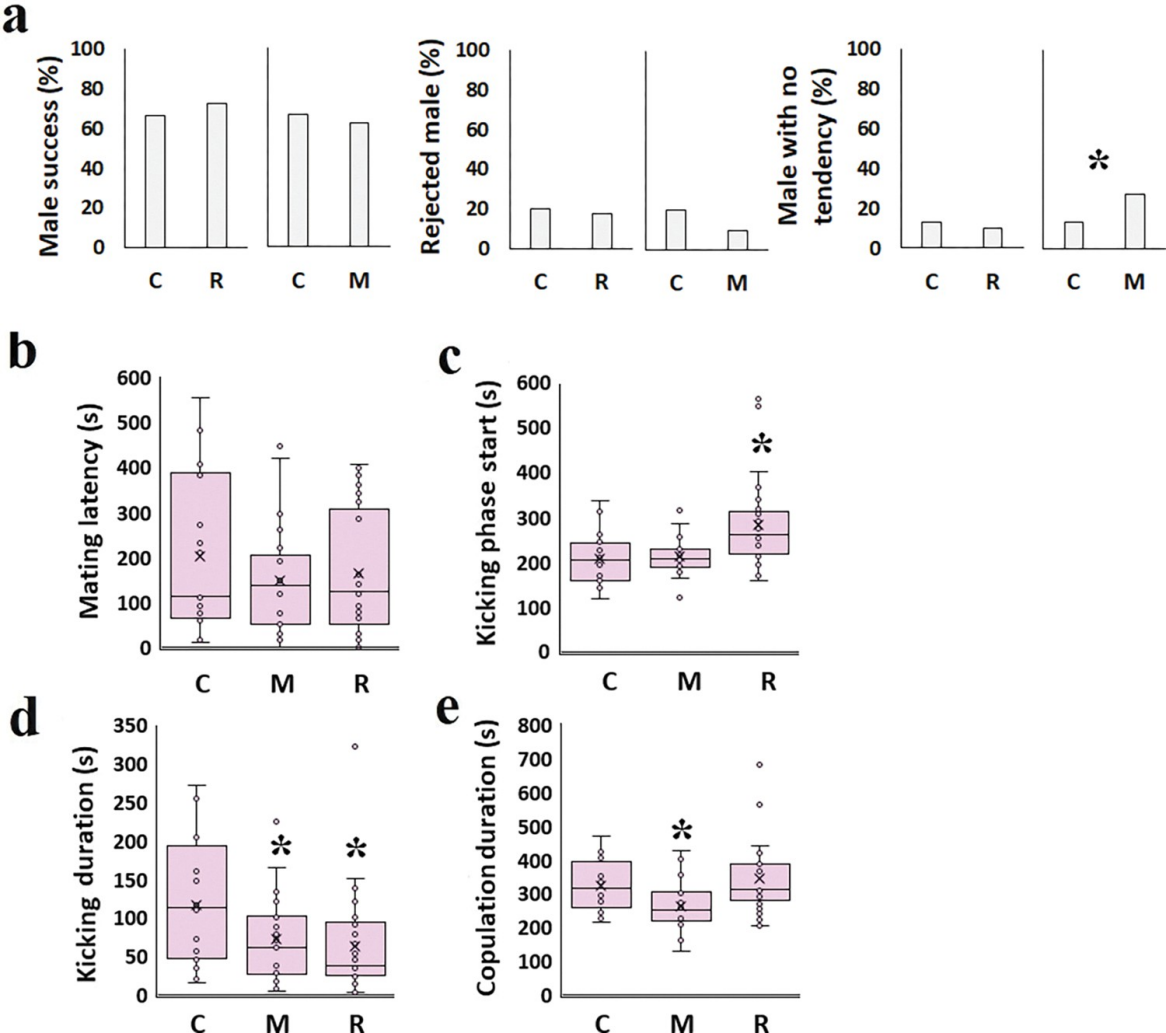
Effect of *C*. *maculatus* exposure to rosemary and mint essential oil in adulthood on copulatory traits of F0 generation. a: Successful mating (%); b: Mating latency (s); c: Kicking phase start (s); d: Kicking duration (s); e: Copulation duration (s). C: No essential oil exposure; M: Mint essential oil exposure; R: Rosemary essential oil exposure. Asterisks represent statistical significance (P<0.05).

Essential oil exposure in adulthood adversely changed AEO insects’ mating behaviors (Fig 4A–4e). For example, mint essential oil-exposed males (M) were more reluctant (27.5%) to initiate mating than the control group (13.3%, χ^2^ = 5.488; df = 1; P = 0.019; Fig 4A). When mating started, females influenced by R started kicking their mate later (281.4±20.3 s, df = 72, t = 3.6, P< 0.0001), and kicking duration was shorter (63.3±19.5 s, df = 72, t = -2.7, P = 0.010) than control (207.4±15.6 s, t = 13.3 and 115.3±15 s, t = 7.7 respectively; Fig 4C and 4D). Also, M decreased kicking duration (72.5±20 s, df = 72, t = -2.1, P = 0.035) and copulation duration (262.5±26.3s, df = 72, t = -2.3, P = 0.025; Fig 4D and 4E) compared with control (115.3±15 s, t = 7.7, and 322.7±19.9 s, t = 16.3, respectively).

### F1 generation

Embryo rosemary and mint essential oil exposure in the F0 generation shifted the F1 sex ratio (female/male) toward fewer females (S1b Fig in S1 File). Also, it slowed down the emergence of F1 adults (47.7±4.1, df = 14, t = -2.3, P = 0.040) compared to the control group (57.1±3.0, t = 18.5, S1c Fig in S1 File) and 76.2% of F1 insects were able to mate successfully when their parents were exposed to rosemary essential oil, compared to 88.1% in the control group (S2 Fig in S1 File).

Parents’ essential oil exposure in adulthood did not modify biological parameters in the F1 generation (S1 Fig in S1 File). These offspring were the same as the offspring of control parents in traits including immature development, sex ratio, adult emergence percentage, adult longevity, egg number, and hatch percentage. However, they had 10% more mating success in the M treatment (S3a Fig in S1 File).

Other mating behaviors such as mating latency, kicking phase start, kicking duration, and copulation duration were not influenced in EEO and AEO F1 generation (S2b-S2e, S3b-S3e Figs in S1 File).

### Biological parameters of F2 generation in embryonic exposure

To know the effect of grandparents’ essential oil exposure on grand offspring performance in a similar environmental condition, we let the F2 generation of both control and essential oil-exposed treatment groups experience the same essential oil as their grandparents. The terms CR and CM referred to control grandparents’ F2 generation exposed to R and M, respectively. Also, RR and MM introduce grand-offspring exposed to the same essential oil as their grandparents (R and M, respectively).

**Fig 5:**
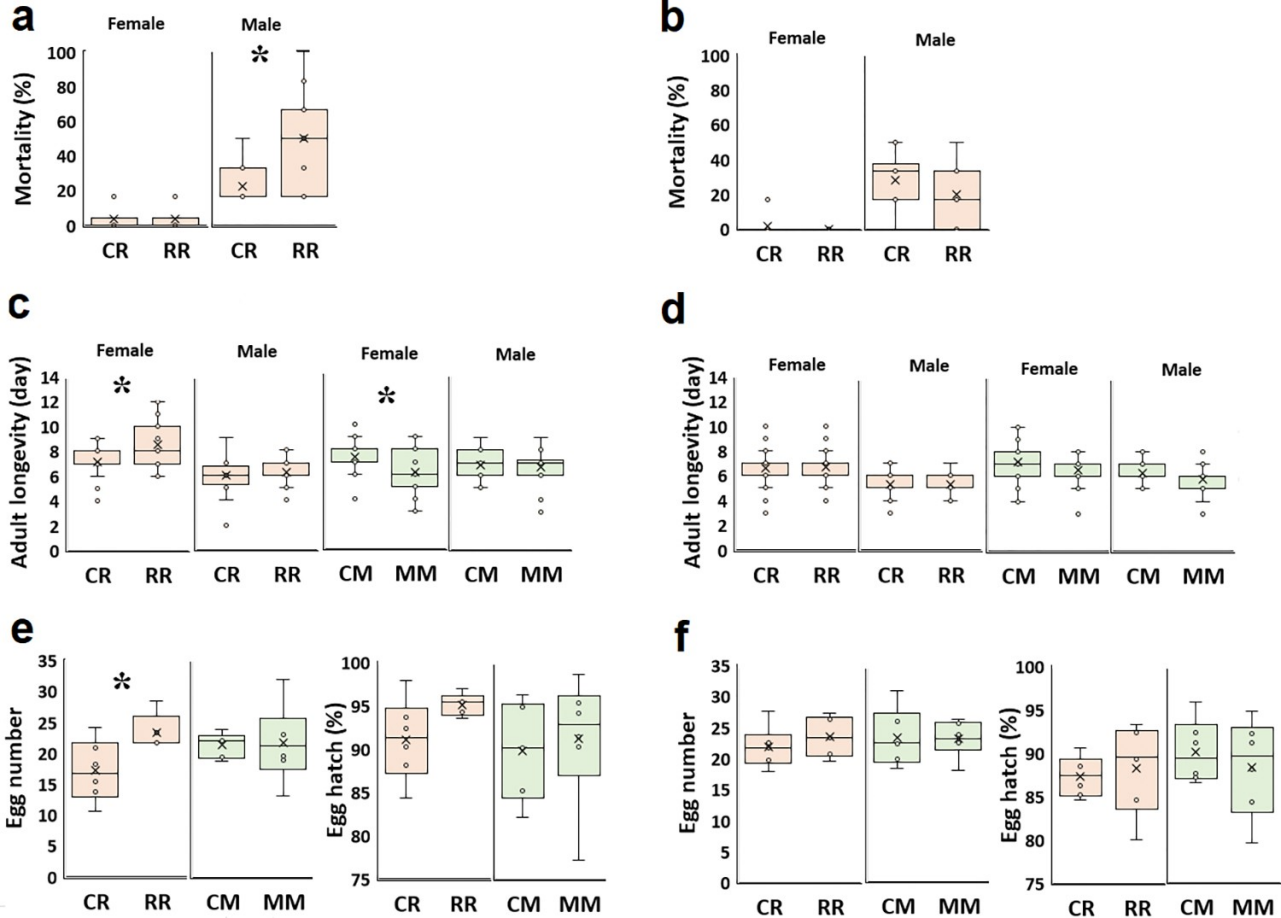
Effect of rosemary and mint essential oil *C*. *maculatus* F2 generation’s biological parameters when facing the same essential oil as their grandparents. a: Mortality (%) in EEO; b: Mortality (%) in AEO; c: Adult longevity (day) in EEO; d: Adult longevity (day) in AEO; e: Egg number and hatch (%) in EEO; f: Egg number and hatch (%) in AEO. CR: F2 of control that treated with rosemary essential oil; RR: F2 of R treatment that treated with rosemary essential oil; CM: F2 of control that treated with mint essential oil; MM: F2 of M treatment that treated with mint essential oil. Orange and green colors represent rosemary and mint essential oil, respectively. Asterisks represent statistical significance (P<0.05).

Having grandparents who experienced the same essential oil in the embryo stage (EEO) improved or deteriorated some traits in the F2 generation when facing a similar essential oil (Fig 5). For example, RR males had a higher mortality percentage 24 hours post-essential oil exposure than CR males (50.0±9.3%, df = 200, t = 2.9, P = 0.008, and 27.7±6.6%, t = 3.4, respectively; Fig 5A).

Also, RR females lived longer than CR females (8.5±0.5 days, df = 62, t = 2.5, P = 0.032, and 7.1±0.3 days, t = 18.8, respectively; Fig 5C). However, the opposite was true of mint essential oil-exposed females. So, MM females lived shorter than CM females (6.2±0.4 days, df = 65, t = -3.1, P = 0.003, and 7.4±0.2 days, t = 27.6, respectively; Fig 5C).

Similar to what we observed in F0, R exposure reduced egg number in CR, though egg number reduction did not occur in RR (CR:17.1±1.7 eggs, df = 14, t = 10.0, and RR: 23.3±2.5 eggs, t = 2.4, P = 0.036; Fig 5E). To have grandparents who had experienced the same essential oil improved the RR offspring’s ability to cope better with that essential oil and maintain egg quantity. This fitness achievement was different based on the essential oil type. For example, the mint essential oil did not raise the F2 generation’s ability to prevent egg number reduction if facing the same essential oil (Fig 5E). None of the treatments altered the egg hatch percentage (Fig 5E).

### Mating behaviors of F2 generation in embryonic exposure

**Fig 6:**
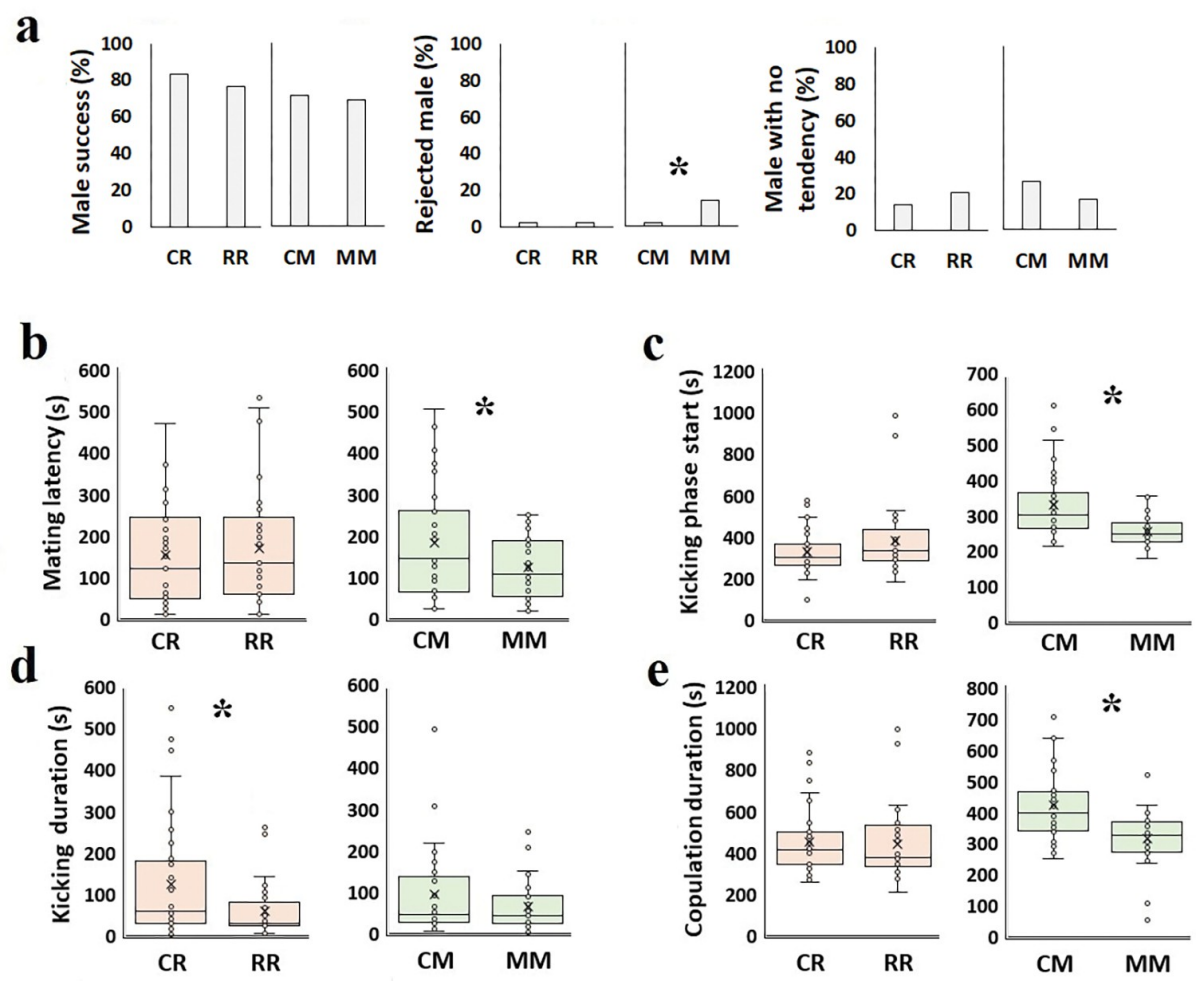
Effect of rosemary and mint essential oil on the EEO *C*. *maculatus* F2 generation’s copulatory traits when facing the same essential oil as their grandparents. a: Successful mating (%); b: Mating latency (s); c: Kicking phase start (s); d: Kicking duration (s); e: Copulation duration (s). CR: F2 of control that treated with rosemary essential oil; RR: F2 of R treatment that treated with rosemary essential oil; CM: F2 of control that treated with mint essential oil; MM: F2 of M treatment that treated with mint essential oil. Orange and green colors represent rosemary and mint essential oil, respectively. Asterisks represent statistical significance (P<0.05).

EEO altered F2 generation copulatory behavior in case of the same essential oil exposure. MM females forcefully prevented male mating efforts, and 14.3% of males’ mating efforts were rejected compared to 2.4% in CM (χ^2^ = 9.0; df = 1; P = 0.003; Fig 6A). By contrast, MM males were more willing to copulate than CM males. Also, MM males had less mating latency (122.5±28.9 s, df = 62, t = -2.0; P = 0.045) than CM (181.7±20.1 s, t = 9.0; Fig 6B). After mating began, MM females started to kick their mate sooner (247.4±21.8 s, df = 62, t = -3.7; P = 0.001) than CM females (327.7±15.1 s, t = 21.6; Fig 6C) to terminate copulation. RR had a shorter kicking duration (60.0±22.1 s, df = 62, t = -2.2; P = 0.035) than CR (122.8±19.6 s, t = 6.3; Fig 6D). Finally, MM had a shorter copulation duration (313.3±27.7 s, df = 62, t = -3.9; P< 0.001) than CM (420.7±19.2 s, t = 21.9; Fig 6E).

### F2 generation of adult exposure

**Fig 7:**
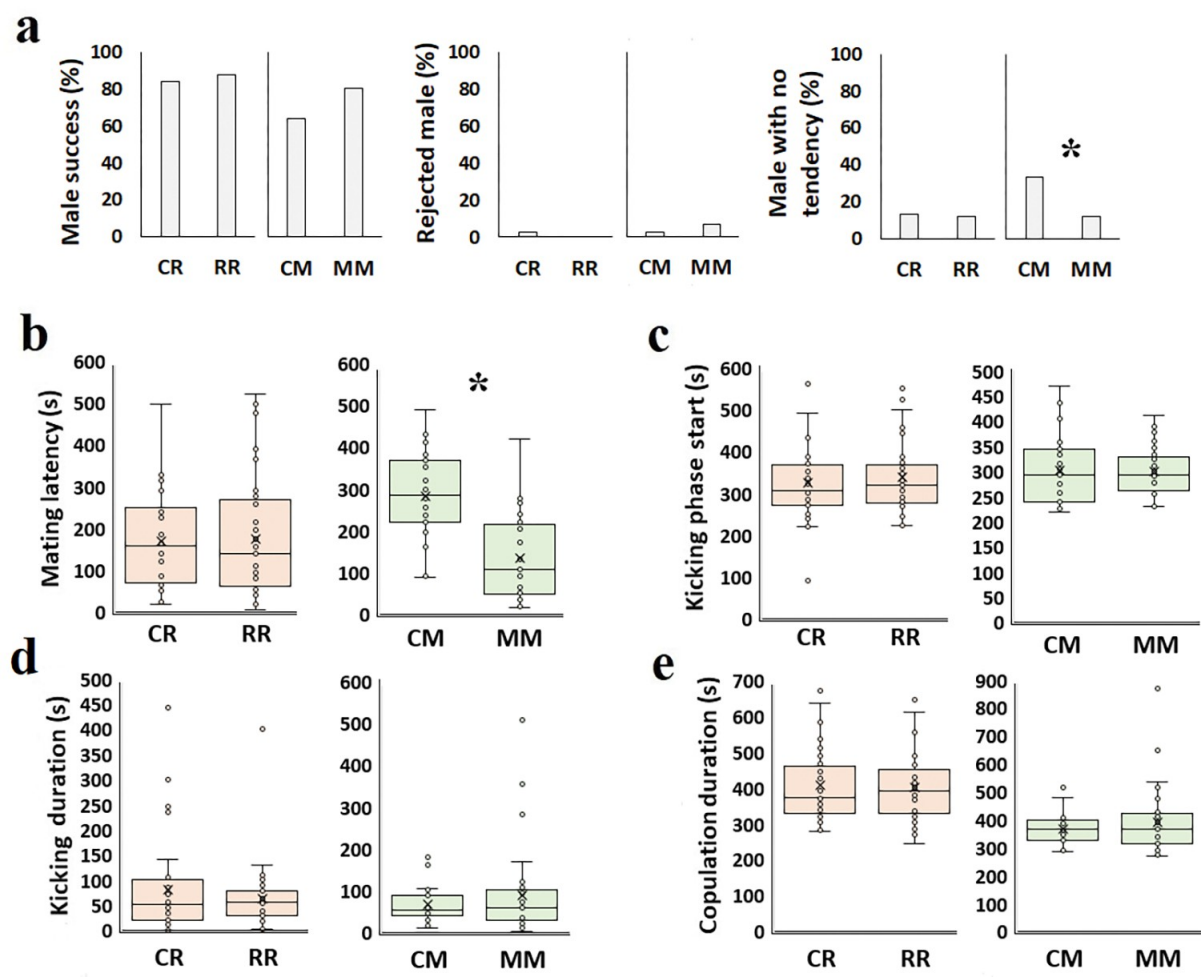
Effect of rosemary and mint essential oil on the AEO *C*. *maculatus* F2 generation’s copulatory traits when facing the same essential oil as their grandparents. a: Successful mating (%); b: Mating latency (s); c: Kicking phase start (s); d: Kicking duration (s); e: Copulation duration (s). CR: F2 of control that treated with rosemary essential oil; RR: F2 of R treatment that treated with rosemary essential oil; CM: F2 of control that treated with mint essential oil; MM: F2 of M treatment that treated with mint essential oil. Orange and green colors represent rosemary and mint essential oil, respectively. Asterisks represent statistical significance (P<0.05).

AEO parents’ F2 generation did not show any additive susceptibility when experiencing the same essential oil (R) or even demonstrated improved features (M stressor). All tested biological parameters (mortality, adult longevity, egg number, and egg hatch percentage) and copulation behaviors of CR and RR were similar (Figs 5 and 7).

In the F2 generation of AEO, 80.9% of MM males copulated successfully compared to 64.1% of CM males (Fig 7A). Also, these males had shorter mating latency (131.9±27.0 s, df = 67, t = -5.5; P< 0.001) compared to CM males (281.9±20.7 s, t = 13.6; Fig 7B). However, other mating behaviors such as kicking phase start, kicking duration, and copulation duration were similar in both the control and treatments groups (Fig 7C–7E).

In all, data shows that in the F0 and F2 generations, insects experienced environmental stressors, and the stressors effect and interaction of stressors and life stage were significant (Table 2).

**Table 2:** Multivariate General Linear Model with Wilks’ Lambda multivariate test in *C*. *maculatus* different generations.

Generation	Factor	Value	F	Hypothesis df	Error df	Sig.
F0	Intercept	0.55	555.27	4	130	<0.001
	Life Stage	0.968	1.07	4	130	0.373
	Stressors	0.842	2.91	8	260	0.004*
	Life Stage*Stressor	0.857	2.59	8	260	0.010*
F1	Intercept	0.37	1758	3	202	<0.001
	Life Stage	0.948	3.659	3	202	0.013*
	Stressors	0.961	1.353	6	404	0.232
	Life Stage*Stressor	0.977	0.795	6	404	0.574
F2	Intercept	0.061	1208.18	3	237	<0.001
	Life Stage	0.977	1.90	3	237	0.13
	Stressors	0.810	5.801	9	576.9	<0.001*
	Life Stage*Stressor	0.920	2.224	9	576.9	0.019*

Asterisks represent statistical significance (P<0.05).

## Discussion

Transgenerational effects are common, robust, and long-lasting, and they can significantly affect organism responses to changing environments [3–5, 22]. The stresses organisms are going through may influence offspring born later and grand offspring [1, 2, 14]. Our data indicated F0 exposure to essential oils induced positive and negative effects in F1 and F2 generations based on essential oil type. Positive and negative insecticide-induced changes might be adaptive, as they can be passed down to future generations and persist across generations due to transgenerational effects [44–46]. While insecticide stress is generally detrimental to fitness, it can occasionally be beneficial and boost fitness [8, 47, 48]. Transgenerational hormesis is defined as the positive effects of a sublethal insecticide exposure of the parental generation on the progeny [49]. For example, sublethal pyrethroid insecticide exposure had a positive within- and across-generations effect on the Colorado potato beetle (*Leptinotarsa decemlineata*). Also, offspring insecticide tolerance increased after maternal insecticide exposure, exhibiting higher larval and pupal survival rates and being heavier than those descending from control mothers [8].

We observed that males and females in the F2 generation responded differently to grandparental effects. For example, in EEO treatment, rosemary essential oil-exposed grandparents produced more tolerant female grand offspring with better ability to cope with the same essential oil (increased adult longevity and egg number). However, male grand offspring were more sensitive (more mortality percentage and less copulation success). The sex of both the parent and the offspring could play a crucial role in driving the evolution of intergenerational plasticity in both adaptive and non-adaptive manners [16]. Parental effects might differ in their influence on male and female offspring. Parental environments, for example, can have opposing effects on the same feature in sons versus daughters or can affect different traits in sons compared to daughters [50–52]. Previous studies have demonstrated that parental experiences activate diverse gene expression and different developmental programs in male and female offspring [46, 53].

The possibility of sex-specific parental effects was examined in a freshwater population of three-spined sticklebacks, *Gasterosteus aculeatus*, by testing offspring antipredator behaviors and brain gene expression after exposing parents to visual cues of predation risk. Results showed that predator-exposed males gave birth to more risk-tolerant male offspring, whereas predator-exposed females gave birth to more risk-averse offspring of both sexes. There were also significant sex-specific effects on brain gene expression [16].

Based on our data, grandparental effects were more robust if grandparents were exposed in the embryonic stage than in adulthood. At the earliest stages of life, environmental variables can produce irreversible developmental changes with long-term performance implications. Some research has indicated that early-life conditions can significantly impact reproductive success years or even decades later [54, 55]. Adult behavior is influenced by early-life stimuli that modify the nervous system’s structure and/or function [56].

Based on our data, parents’ exposure to essential oils influenced F1 offspring copulation behaviors in both embryonic and adulthood-treated parents. Animal behavior is frequently viewed as the most plastic component of phenotypes that parental effects can modify [6, 18, 57]. For example, the *C*. *maculatus* embryo, when exposed to eucalyptus leaf and flower essential oils, compensated for negative consequences by altering reproductive behavior and a trade-off between fecundity and longevity. Essential oil effects remained until the F1 generation, which had not been exposed to essential oil directly. The F2 generation, on the other hand, was not influenced by their grandparents’ experiences [31]. *Myzus persicae* aphids treated with sublethal amounts of imidacloprid produce progeny that survive longer when exposed to food/water stress, but their tolerance to pesticide stress remains constant [58]. Although precocene (a juvenile hormone antagonist) stimulates reproduction in *M*. *persicae* at sublethal levels, the effects are not passed down to later generations [59].

The present study showed grandparental effects could influence grand offspring’s reactions when encountering the same essential oils that their grandparents had already experienced. Environmental variation experienced in previous generations can have a complex and difficult-to-predict impact on progeny phenotypes. In some circumstances, parental environments affect the phenotypes of the F1 generation, but these effects do not last into subsequent generations (e.g., F2) [14, 31, 59]. It has been documented that when one generation experiences a situation, the effect of that experience could be found in the next generations, even if offspring were not raised in parents’ or grandparents’ conditions (grandparental effects) [46, 53, 60]. In *Drosophila serrata*, the impact of parental and grandparental exposure to a non-lethal cold shock was studied. Development time is frequently slowed down following maternal and/or grandmaternal cold exposure. The effects of parental cold exposure on viability were unfavorable, but the impact of grandparental cold exposure on viability was minimal. Mothers’ cold exposure enhanced offspring productivity, but grandmothers’ cold exposure decreased it. Cold shock exposure in male parents and grandparents reduced male productivity [61].

Based on our data, rosemary essential oil could be more effective than mint essential oil for pest control. However, we also found that when F0 *C*. *maculatus* experienced sublethal rosemary essential oil in the embryonic stage, the next generations could better cope with the same stressor. Therefore, long-term use of this essential oil in the field for pest control mitigates the intended efficacy of the oil in suppressing pest populations over time.

In conclusion, *C*. *maculatus* life experiences of embryonic or adult exposure to rosemary or mint essential oils had lasting consequences for the F1 and F2 generations. When grandparents were exposed to a stressor in the embryonic stage, more intense grandparental effects were observed in F2 compared to grandparents’ stress experiences in adulthood. Also, the sex of grand offspring and the stressor type were effective in improving the quality of grandparental effects. Female grand offspring showed adaptive responses to rosemary essential oil and non-adaptive responses to mint essential oil. It could be because of the different modes of action of these essential oils, and more studies are needed. In all, stressor type and sex of grand offspring are important in *C*. *maculatus* grandparental effects.

## Supporting information

S1 File (DOCX)

S1 Data Data files. (ZIP)
