# Supplementary material for: Callosobruchus maculatus males and females respond differently to grandparental effects
Source: PLoS One. 2023 Dec 22;18(12):e0295937. doi: 10.1371/journal.pone.0295937 (PMC10745144; doi:10.1371/journal.pone.0295937)
Supplement: S1 File — (DOCX) [file pone.0295937.s001.docx]

***Callosobruchus* *maculatus* males and females respond differently to grandparental effects**

Azam Amiri^*1^ and Ali R. Bandani^2^

1. College of Geography and Environmental Planning. University of Sistan and Baluchestan, Zahedan, Iran.

2. Department of Plant Protection, College of Agriculture and Natural Resources, University of Tehran, Karaj, Iran. Email: abandani@ut.ac.ir

^*^Corresponding author email: [azamamiri@eco.usb.ac.ir](mailto:azamamiri@eco.usb.ac.ir)

Corresponding author Orcid ID: 0000-0003-2709-0061

**Supplementary data**

Supplementary Table 1. Summary of statistical analysis.

| Factor | Statistical tests |
| --- | --- |
| Immature development time, adult longevity, sex ratio, adult emergence, egg number, egg hatch, mating latency, kicking phase start, kicking duration, copulation duration | Linear Mixed Model; Tested factor as fixed effect + Petri dish  as random effect |
| Male success, rejected male, male with no tendency | Chi-square goodness-of-fit tests |
| Life Stage, Stressors, Life Stage*Stressor | Multivariate General Linear Model with Tukey’s post hoc Multiple comparisons, Bonferroni confidence interval adjustment, Wilks’ Lambda multivariate |


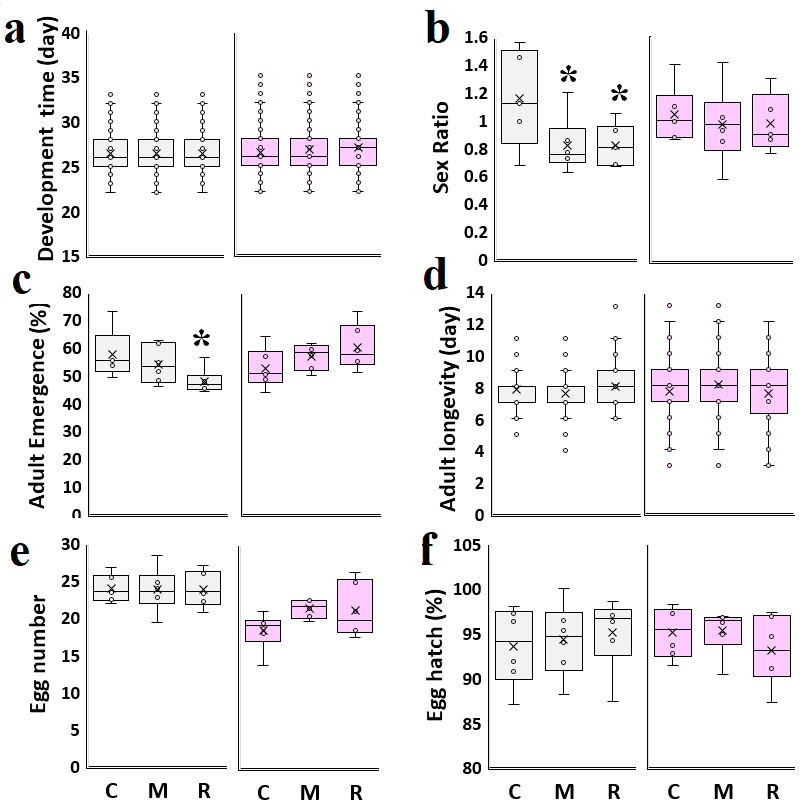


Supplementary Fig. 1. Effect of F0 essential oil exposure on different biology parameters of *C. maculatus* F1 generation. a: Immature development time (day); b: Sex Ratio (Female/Male); c: Adult emergence (%); d: Adult longevity (day); e: Egg number; f: egg hatch (%). C: No essential oil exposure; M: Mint essential oil exposure; R: Rosemary essential oil exposure. Grey and pink colors presented EEO and AEO conditions. The sex ratio of offspring in EEO parents decreased toward less female offspring production. Statistical signiﬁcance was established as P<0.05.


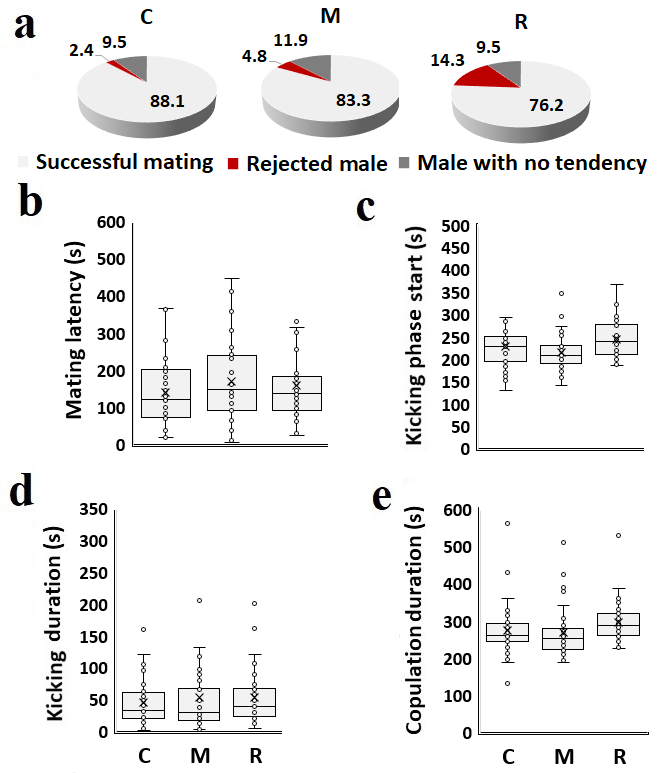


Supplementary Fig. 2. Effect of *C. maculatus* F0 exposure to the essential oil in embryonic stage on copulatory traits of the F1 generation.

a: Successful mating (%); b: Mating latency (s); c: Kicking phase start (s); d: Kicking duration (s); e: Copulation duration (s). C: No essential oil exposure; M: Mint essential oil exposure; R: Rosemary essential oil exposure. Statistical signiﬁcance was established as P<0.05.


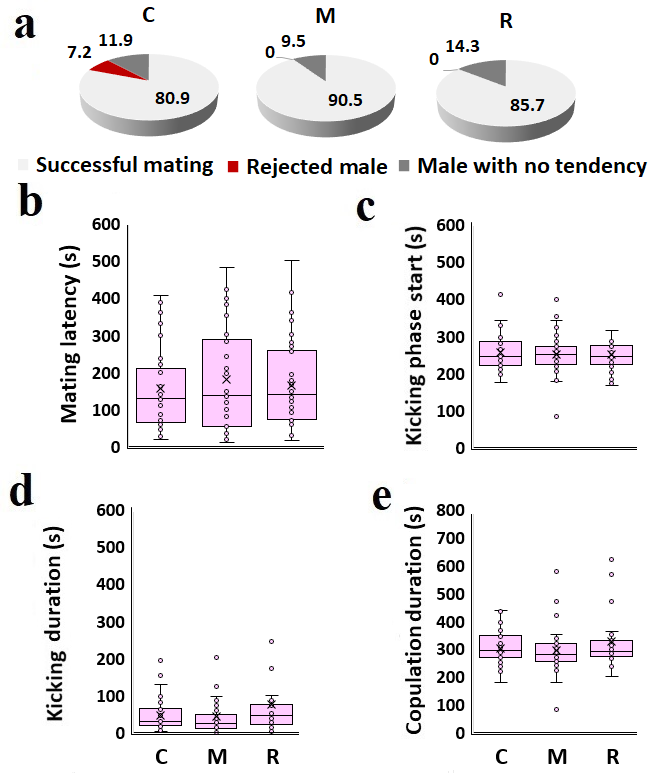


Supplementary Fig. 3. Effect of *C. maculatus* F0 exposure to the essential oil in adulthood on copulatory traits of the F1 generation.

a: Successful mating (%); b: Mating latency (s); c: Kicking phase start (s); d: Kicking duration (s); e: Copulation duration (s). C: No essential oil exposure; M: Mint essential oil exposure; R: Rosemary essential oil exposure. Statistical signiﬁcance was established as P<0.05.
